# Influence of Body Mass Index and Albumin on Perioperative Morbidity and Clinical Outcomes in Resected Pancreatic Adenocarcinoma

**DOI:** 10.1371/journal.pone.0152172

**Published:** 2016-03-25

**Authors:** Andrew Hendifar, Arsen Osipov, Jasleen Khanuja, Nicholas Nissen, Jason Naziri, Wensha Yang, Quanlin Li, Richard Tuli

**Affiliations:** 1 Department of Medicine, Cedars-Sinai Medical Center, Los Angeles, CA, United States of America; 2 Department of Surgery, Cedars-Sinai Medical Center, Los Angeles, CA, United States of America; 3 Department of Radiation Oncology, Cedars-Sinai Medical Center, Los Angeles, CA, United States of America; University of Florida, UNITED STATES

## Abstract

Obesity is a known risk factor for PDA and recent reports suggest obesity has a negative impact on clinical outcomes in patients with PDA. Pretreatment body mass index (BMI) and serum albumin (SA) have been shown to be associated with worse overall survival in patients with advanced and metastatic PDA. However, minimal data exists on the impact of BMI and SA on perioperative and long-term clinical outcomes in patients with early-stage resected PDA. Herein, we report on the impact of these variables on perioperative clinical outcomes, overall survival (OS) and disease free survival (DFS) in patients with resected PDA. With IRB approval, we evaluated 1,545 patients with PDA treated at a single institution from 2007–2013 and identified 106 patients who underwent upfront resection with curative intent. BMI and SA were calculated preoperatively and at the time of last clinical evaluation. Influence of preoperative BMI, SA, change in either variable, and influence of other clinical and pathologic variables on perioperative morbidity and mortality was assessed. The impact of these variables on DFS and OS was assessed with cox regression modeling and ANOVA. Actuarial estimates for DFS and OS were calculated using Kaplan-Meier methods. Median follow up time was 16 months (3–89). Mean age was 68 years. Median survival was 14 months (3–65) and median time to recurrence was 11 months (1–79). Length of hospital stay was associated with BMI (p = .023), change in BMI (p = .003) and SA (p = .004). Post-operative transfusion rate was associated with SA (p = .021). There was a strong correlation between BMI change and positive margin (p = .04) and lymph node status (p = .01). On multivariate analysis, change in SA (p = .03) and node positivity (p = .008) were associated with decreased DFS. Additionally, preoperative SA (p = .023), node positivity (p = .026) and poor differentiation (p = .045) were associated with worse OS on multivariate analysis. Low preoperative SA was associated with worse DFS and OS in patients with resected PDA. Lower BMI and SA were associated with longer post-operative hospital stay. Our study is one of the first to describe how pre-operative BMI and SA and post-operative changes in these variables impact clinical and perioperative outcomes. This data supports nutritional status and weight loss as predictors of outcome in resected pancreatic cancer patients and warrants further prospective investigation.

## Introduction

Pancreatic ductal adenocarcinoma (PDA) is the 4^th^leading cause of cancer death[1]. Despite advances in the treatment and management of this malignancy, 5-year survival is still only 6%. Patients who have early stage disease are often those with the best outcomes[2, 3]. Therefore, increased attention has been paid to known risk factors for this disease including family history [4, 5], diabetes [6, 7] and obesity.

Obesity is a known risk factor for the development of PDA [8–12]. Obesity associated PDA has also been linked with decreased physical activity [8, 13] and younger age of onset [14]. Recent reports also suggest that obesity has a negative impact on outcomes in patients with a known diagnosis of PDA [15]. However, a mechanistic explanation for the association between obesity and pancreatic cancer development remains under investigation.

A recent meta-analysis has shown that pretreatment serum albumin (SA) is also prognostic of outcome in patients receiving anti-cancer therapy for PDA [16]. Whereas prediagnostic body mass index (BMI) and SA have been shown to be associated with decreased overall survival in patients with advanced disease, it’s impact on perioperative and long-term clinical outcomes in patients with early stage resected PDA have not been thoroughly evaluated. We therefore assessed the impact of BMI and SA on perioperative clinical outcomes, overall (OS) and disease-free survival (DFS) in patients with resected PDA.

## Methods

With Cedars-Sinai Medical Center Institutional Review Board approval, we evaluated the medical records of 1,545 PDA patients treated at our institution from 2007–2013. Of these, we identified 106 patients with long-term follow up who underwent resection with curative intent. Patient records and information were anonymized and de-identified prior to analysis. Patients with ampullary, duodenal, distal bile duct, neuroendocrine, and cystic neoplasms were excluded. Patient follow-up was obtained through office and electronic medical records and retrieval of death certificates of patients living within the USA. Clinical and pathologic variables assessed included patient demographics, stage, nodal involvement, margin status, tumor grade, and type of adjuvant therapy (Table 1).

**Table 1:** Patient Disease Specific Characteristics.

Variable/Characteristic	N	Percent (%)	Mean
**Age**			
			68
**Sex**			
Male	54	51	
Female	52	49	
**Race**			
Caucasian	79	74.5	
African American	5	4.7	
Asian	11	10.4	
Hispanic	11	10.4	
**Location**			
A. Uncinate, Head Neck	90	84.9	
B. Body, Tail	15	14.2	
Combined (A+B)	1	0.1	
**Type of Resection**			
Pancreaticoduodenectomy	93	87.7	
Distal Pancreatectomy	13	12.3	
**T Stage**			
T1	2	1.9	
T2	10	9.4	
T3	91	85.9	
T4	3	2.8	
**N Stage**			
N0	34	32.1	
N1	72	67.9	
**Margin Distance**			
positive (tumor at ink)	18	17.0	
≤ 1 mm	50	47.2	
> 1 mm	38	35.8	
**Adjuvant Therapy**			
Chemotherapy	51	48.1	
Chemoradiation	45	42.5	
No Adjuvant Therapy	10	9.4	
**Periportal Lymphadenectomy**			
Yes	25	23.6	
No	81	76.4	
**Periportal Lymph Nodes**			
≥ 1	5	20	
None	20	80	
**Perineural Invasion**			
Present	94	92.2	
Not Present	8	7.8	
**Lymphvascular invasion**			
Present	59	59	
Not Present	41	41	
**Tumor Grade**			
1	17	16.5	
2	52	50.5	
3	34	33.0	

Immediate pre-operative, 30-day post-operative and last recorded BMI and SA values were assessed and used to calculate BMI- and SA-change, respectively. Pre-operative BMI was categorized as follows: < 19, 19–29, >/ = 30. BMI change was defined as the difference between the last BMI measured and the BMI at surgery (BMI (last known)–BMI (surgery)). The same methodology was used to calculate change in SA. Pre-operative SA was categorized as < 3.5 or >/ = 3.5. SA was also evaluated as a continuous variable.

Perioperative variables assessed included total operating time, intraoperative blood loss, transfusion requirement and length of stay. Rehospitalization rates and perioperative mortality were calculated at 30 and 60 days post-operatively. DFS was defined as the last date the patient was known to be alive and without clinical or radiographic evidence of recurrence. The impact of these variables on 30- and 60-day rehospitalization and/or mortality, DFS and OS was assessed using log-rank test and Cox proportional hazards model. Multivariable Cox regression was also used to adjust for confounders when assessing a predictor of interest. Association between variables was based on logistic regression or two sample t tests. P value < 0.05 was considered statistically significant.

## Results

The median follow up time for the 106 resected PDA patients was 16 months (2.5–89). Forty nine percent (n = 52) were women. Patient ethnicities in this analysis included Caucasian (75%), Hispanic (10%), Asian (10%) and African American (5%). The mean age of the patients was 68. Patients were categorized as having primary tumors in the proximal (head/neck/uncinate) or distal pancreas (body/tail). The majority of patients had a proximal lesion (85%) and 88% underwent a pancreaticoduodenectomy, as opposed to a distal pancreatectomy. Most patients (85%) had T3 disease (N = 90) and 66% were node positive (n = 35) with 64% having stage 2B disease (n = 68). Margin positivity defined as tumor at ink was identified in 17% (n = 18). The number of positive nodes resected ranged from 0–21 with a median of 19 nodes resected and 2 nodes positive. Additional clinical and pathologic data is included in Table 1. Among patients who expired (N = 44), the median survival time was 14.1(3.4–64.7) months. Median time to recurrence was 11.1 months (0.9–78.3) months.

Of the 106 patients included in the study, pre-operative BMI was < 19, 19–29, and >/ = 30 in 11, 77 and 12 patients, respectively. Pre-operative SA was < 3.5 or >/ = 3.5 in 57 and 40 patients, respectively. Either pre-operative or post-operative data for BMI and SA was not available for 6 and 9 patients, respectively. Median pre-operative BMI was 24.1 (14–47.9) and the median change was -1.6. Median pre-operative SA was 3.3 (1.7–4.9) and median change was -0.4. Median operation time was 8.1 hours (3.1–15.7) and blood loss was 500 cc (100–1500). Median hospital stay was 8 days (3–90). Median units of transfused blood was 0 (0–5).

### Peri-Operative Rehospitalization and Morbidity

Twenty-one patients (20%) were hospitalized within 30 days of pancreatic surgery. Following univariate analysis, no reliable predictors for re-hospitalization within 30 days were identified. Specifically, blood loss, operative time, age, and pre-operative SA and BMI were not associated with re-hospitalization. Rehospitalization within 30 days was not associated with worse DFS or OS. No patients died within the 30 or 60-day post-operative period

Notably, very few peri-operative outcomes were associated with pre-treatment SA, BMI or changes in either variable. However, length of hospital stay was associated with low BMI (p = 0.023), low SA (p = 0.004), and a reduction in BMI (p = 0.003). Post-operative transfusion rate was also associated with SA (p = 0.021) but not BMI. The length of the operation was associated with neither factor. Interestingly, there was a strong correlation between BMI change and positive margin (p = 0.04) and lymph node status (p = 0.01). SA was not associated with margin status, lymph node status, histologic grade or receipt of adjuvant therapy. Whereas no association between BMI and SA, or change in BMI to change in SA was identified, strong associations between pre-treatment BMI and change in BMI (rho = -0.5, p<0.001), and SA and change in SA were found (rho = -0.56,p<0.001).

### Disease-free and overall survival analysis

**Fig 1:**
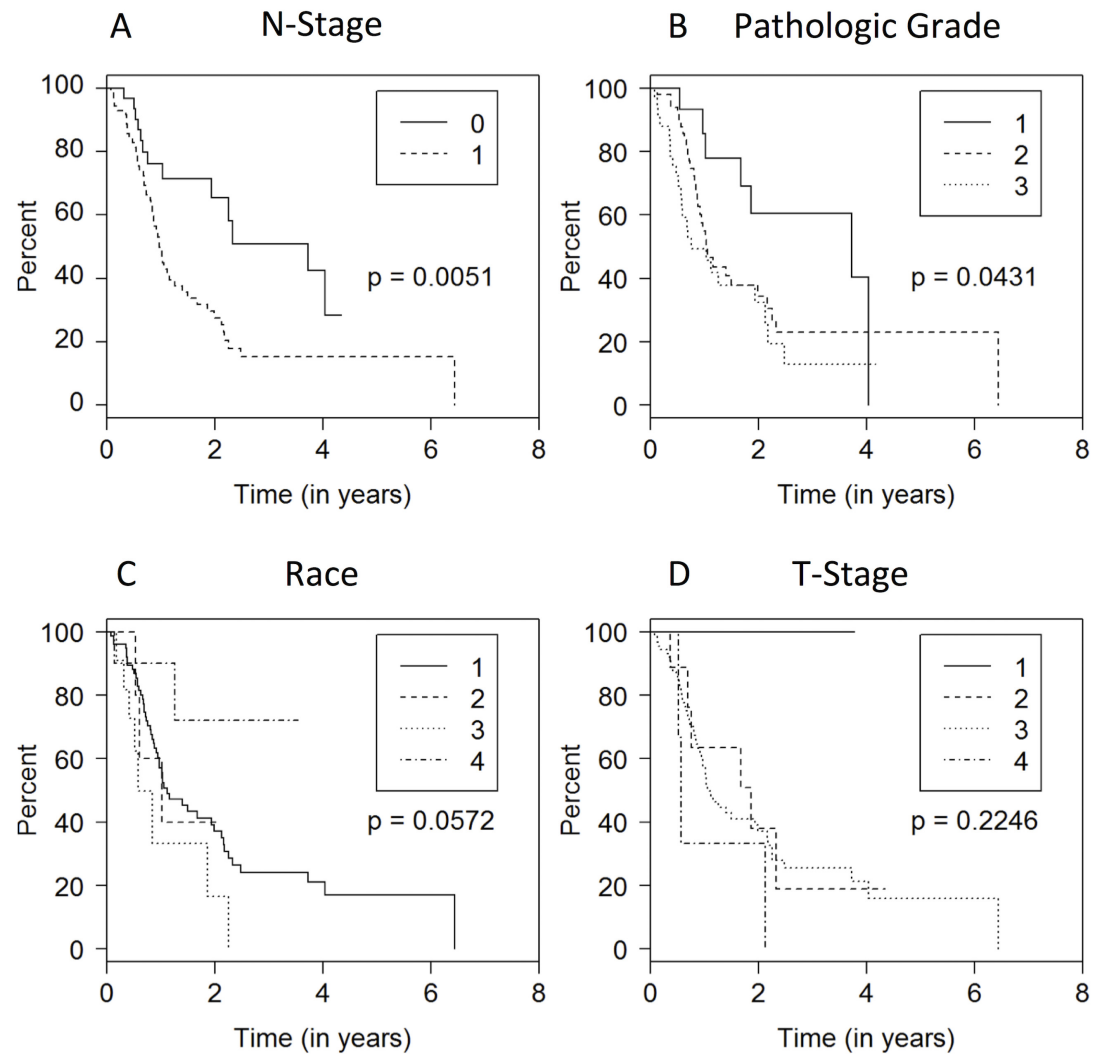
Patient and disease factors associated with disease free survival. (A) Disease Free Survival with Node Stage 0 (0) vs Node Stage 1 (1). (B) Disease Free Survival of histologic Grade utilizing three tier grading (1: well differentiated, 2: intermediate differentiation, poorly differentiated). (C) Disease Free Survival Among various races (1: Caucasian, 2: African American, 3: Asian, 4: Hispanic). (D) Disease Free Survival Across Tumor Stage (T1-T4).

Following univariate analysis, nodal involvement and histologic grade were associated with disease-free survival (DFS; p<0.05; **Fig 1**), while pre-treatment CA 19–9, race, and pathologic tumor size were borderline significant predictors of inferior DFS. Patients with nodal disease had decreased DFS (HR 2.36, P = 0.006), as did those with poorly differentiated tumors (HR 2.83, P = 0.017). Interestingly, neither BMI, albumin, nor change in either parameter was associated with DFS (P = 0.43, 0.30, 0.36, 0.18). Multivariate cox proportional model was utilized to further evaluate predictors of DFS. After controlling for pre-treatment CA 19–9, nodal involvement, change in SA, pathologic stage (T), sex, race, and tumor differentiation, change in SA was associated with DFS. Change in SA, as a continuous variable (p = 0.03), and nodal positivity (p = 0.009) were associated with decreased DFS (**Table 2**). Additionally, a decrease in SA in excess of 0.6 g/dL led to significantly worse DFS (HR = 2.2; CI 1.11–4.37, p = 0.024)

**Table 2:** Parameter Estimates from Multivariate Cox Regression Model for Disease-free Survival.

	Coefficient	Hazard Ratio	*p*-value
Albumin Change	0.171	1.186	0.232
Nodal Positivity	1.077	2.935	0.009
preCA19(log)	0.178	1.195	0.098
PathSize	0.224	1.252	0.096
Race: Africian American	-0.426	0.653	0.573
Race: Asian	0.430	1.527	0.478
Race: Hispanic	-1.803	0.165	0.082

**Fig 2:**
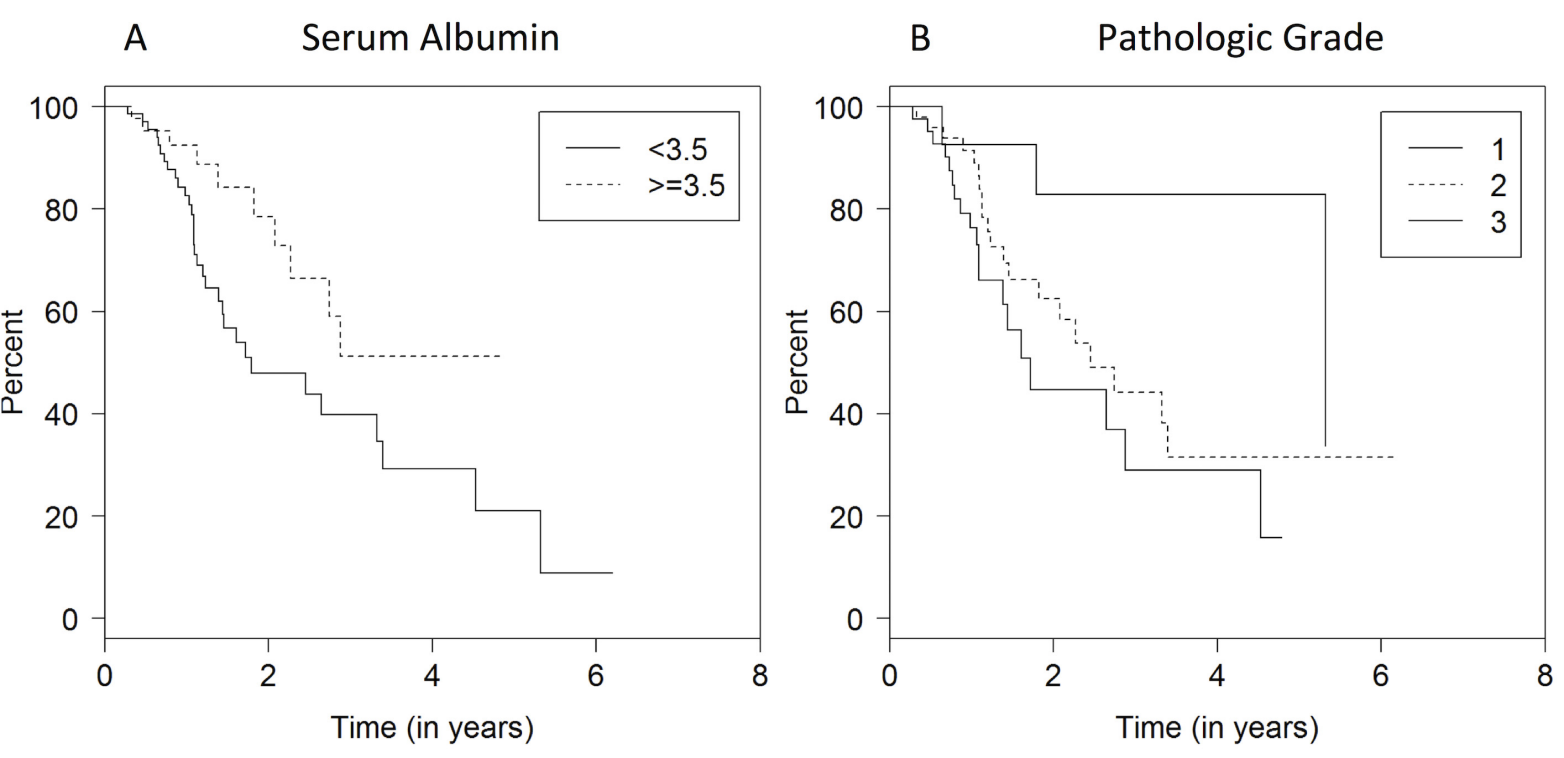
Predictors of increased mortality, SA and histologic grade. (A). Overall survival of patients with SA < 3.5 vs those with SA ≥ 3.5. (B) Overall Survival of Histologic Grade utilizing three tier grading (1: well differentiated, 2: intermediate differentiation, 3: poorly differentiated).

Predictors for increased mortality were also examined using univariate analysis. Patients with pre-operative SA < 3.5 had worse OS when compared to those with a SA ≥ 3.5 (HR 0.48 P = 0.04), as did patients with poorly differentiated tumors compared to well-differentiated tumors (HR 5.44, P = 0.0067) (**Fig 2**). When we examined albumin as a continuous variable we also found a significant association with OS (p = 0.0101). However, pre-operative BMI and BMI change were not associated with worse OS overall.

A multivariate cox proportional model was used to further evaluate the interaction between covariates (**Table 3**). In our multivariate model, preoperative SA when evaluated as either a binary or continuous variable was significantly associated with OS. The number of positive nodes (P = 0.026) and poor differentiation (P = 0.045) were also associated with worse OS.

**Table 3:** Parameter Estimates from Multivariate Cox Regression Model for Overall Survival.

	Coefficient	Hazard Ratio	*p*-value
Preoperative	-0.729	0.483	0.008
Number of Positive Nodes	0.079	1.083	0.026
PathSize	0.171	1.186	0.232
Grade/Differentiation: 2	0.909	2.483	0.154
Grade/Differentiation: 3	1.323	3.753	0.045

## Discussion

We report for the first time that higher pre-operative SA is associated with greater OS in a cohort of patients with resected pancreatic cancer. Additionally, a significant decrease in pre-operative SA led to statistically significant decrease in DFS. This benefit does not appear to be related to quality indicators for peri-operative outcomes including blood loss, margin status, or 30-day re-hospitalization. There was an association between lower SA and transfusion rate and length of hospital stay of questionable clinical significance.

Interestingly, we did not find that an elevated BMI was associated with decreased DFS or OS. Whereas pretreatment BMI, SA and change in these variables had no effect on blood loss, transfusion rate, or 30-day re-hospitalization, they did result in significantly prolonged hospital stays. Additionally, we noted that a reduction in BMI was significantly associated with negative pathologic factors, including positive nodal disease and margins.

The concept that poor nutritional status would be associated with worse outcomes makes intuitive sense. Pre-operative SA is generally considered to be a well-studied and reliable predictor of surgical outcomes [17]. We comprehensively assessed peri-operative clinical outcomes and tumor characteristics but did not find any covariates to explain this relationship further. One would assume that a patient with poor nutrition status would have increased hospitalization rates or increased perioperative morbidity. However, our data suggests that decreased albumin is a poor prognostic factor independent of perioperative morbidity especially in excess 0.6 g/dL. This may potentially reflect more aggressive cancer biology. The relationship between pancreatic cancer cachexia and aggressive tumor biology has been previously reported [18].

Anorexia/cachexia syndrome is a widely described process in pancreatic adenocarcinoma patients [19]. This syndrome is characterized by weight loss, muscle wasting, and poor nutritional status [20]. These patients uniformly have worse outcomes [21]. Unfortunately, there are no therapies to date that have established efficacy for cachexia in this patient population. Muscle loss or sarcopenia is thought to be the worst prognostic indicator in this syndrome. Sarcopenia, as assessed radiographically by total psoas area (TPA) and Hounsfield Unit Average Calculation of the psoas muscle, has been associated with worse outcomes in prostate, renal cell, and pancreatic cancer patients treated with chemotherapy [22–23]. Using lowest quartile TPA as an indicator of sarcopenia in resected pancreatic cancer patients, Peng et al. found it to be independently associated with a 63% increased risk of death at 3 years relative to non-sarcopenic patients [24]. Interestingly and similar to our data with SA and BMI, the authors found no association between sarcopenia and worse perioperative and short-term morbidity. This was hypothesized to be a result of a low absolute rate of such events in the entire cohort. In a follow up to this study, total psoas volume (TPV) was compared to TPA and identified to as an independent predictor of both postoperative complications and long-term survival [25]. Whereas these modalities have not been prospectively validated, they may potentially serve to risk stratify patients in the post-operative setting and aid in selecting appropriate adjuvant treatment strategies. There is emerging evidence that strictly classifying resection margins as negative (R0; no tumor at ink) or positive (R1; tumor at ink) according to the UICC definition may not provide adequate prognostic information as it relates to likelihood of locoregional recurrence [26]. Indeed, previous studies have reported vastly different rates of R1 resections (20–80%) based on the pathologic criteria utilized (Royal College of Pathologists or UICC [26, 27]. In our cohort, resection margins classified utilizing the Royal College of Pathologists and UICC definitions resulted in R1 resection rates of 64% and 17%, respectively. Given the potential prognostic significance of a close margin (<1 mm), we have reclassified this subgroup of patients accordingly [27, 28].

Our study is one of the first to describe how pre-operative BMI and SA and post-operative changes in these variables impact clinical and perioperative outcomes any may be suitable to identify high-risk pancreatic cancer patients. This data supports nutritional status and weight loss as prognostic of outcome in resected pancreatic cancer patients and warrant further prospective investigation.
